# Dissemination of Extended-Spectrum *β*-Lactamase- and AmpC *β*-Lactamase-Producing* Escherichia coli* within the Food Distribution System of Ho Chi Minh City, Vietnam

**DOI:** 10.1155/2016/8182096

**Published:** 2016-02-17

**Authors:** Do Phuc Nguyen, Thi Anh Dao Nguyen, Thi Hien Le, Nguyen Minh Doan Tran, Thanh Phong Ngo, Van Chinh Dang, Takao Kawai, Masashi Kanki, Ryuji Kawahara, Michio Jinnai, Shinya Yonogi, Yuji Hirai, Yoshimasa Yamamoto, Yuko Kumeda

**Affiliations:** ^1^Institute of Public Health, Ministry of Health, 159 Hung Phu Street, Ward 8, District 8, Ho Chi Minh City 700000, Vietnam; ^2^Osaka Prefectural Institute of Public Health, 1-3-69 Nakamichi, Higashinariku, Osaka 537-0025, Japan; ^3^Global Collaboration Center, Osaka University, 2-7 Yamadaoka, Suita, Osaka 565-0871, Japan

## Abstract

To investigate the dissemination of ESBL/pAmpC-producing* E. coli* within the food distribution system of Ho Chi Minh City (HCMC), Vietnam, the prevalence of ESBL/pAmpC-producing* E. coli *strains in chicken meat, pork, beef, and fish/shrimp samples obtained from slaughterhouses, a wholesale market, and supermarkets was examined. Among the total of 330 collected food samples, ESBL/pAmpC-producing* E. coli* was detected in 150 samples (45.5%). The highest prevalence of these isolates was in chicken meat (76/82, 92.7%), followed by pork (32/92, 34.8%), beef (18/74, 34.3%), and fish/shrimp (24/82, 29.3%). A total of 342 strains of ESBL/pAmpC-producing* E. coli* were isolated from 150 positive food samples. The most prevalent genes responsible for ESBL or pAmpC activity belonged to the CTX-M-9 (110/342, 31.2%), CTX-M-1 (102/342, 29.8%), and CIT (118/342, 34.5%) groups. To our knowledge, this is the first report of the high occurrence of pAmpC (37.1%) in animal-based food in Vietnam. Among the 342 total ESBL/pAmpC-producing* E. coli* isolates, 276 (80.7%) were resistant to at least 6 antibiotic agents. Notably, high percentages of resistance to ciprofloxacin and fosfomycin were found in isolates from chicken (80.5% and 50.8%, resp.). These findings demonstrate that animal-based food products in HCMC represent a major reservoir of ESBL/pAmpC-producing* E. coli*.

## 1. Introduction

Strains of extended-spectrum *β*-lactamase- (ESBL-) and plasmid-mediated AmpC *β*-lactamase- (pAmpC-) producing* Escherichia coli* pose a threat to public health because of their ability to hydrolyze third-generation cephalosporins, which are commonly used to treat serious infections caused by members of the Enterobacteriaceae family. Such drug resistance is likely acquired by* E. coli *strains colonizing the gastrointestinal tract of animals, as well as humans, treated with and/or fed antimicrobial agents, including cephalosporins.

Recently, a high prevalence of ESBL-producing* E. coli* was reported in healthy individuals in Southeast Asia, including China (65.0%) [1], Thailand (58.2%–69.3%) [2], and Vietnam (51.0%) [3], and among healthy tourists traveling from the Netherlands to East and South Asia (67% and 72%, resp.) [4]. These colonization percentages suggest that ESBL-producing* E. coli* has the potential to be transmitted from food-producing animals to humans via the food chain. However, the extent to which animal-based food products contribute to the dissemination of ESBL-producing* E. coli* strains to human populations in Southeast Asia has not yet been investigated. Furthermore, no data regarding the prevalence of pAmpC-producing* E. coli* strains in food in Southeast Asia is available.

In the present study, we investigated the prevalence of ESBL- and/or pAmpC-producing* E. coli* strains in chicken, pork, beef, and fish/shrimp samples collected from within the food distribution system of Ho Chi Minh City (HCMC). HCMC was selected because it is the most-populated city in Vietnam and contains the highest number of supermarkets. In addition, HCMC contains large-scale slaughterhouses for poultry, pigs, and cattle and also large wholesale markets for fish and shrimp.

## 2. Materials and Methods

### 2.1. Sampling

The food samples used in this study were collected from 4 slaughterhouses (1 poultry, 2 pig, and 1 beef cattle), a wholesale market (fish/shrimp), and 8 supermarkets in HCMC between October 2012 and March 2014. The poultry slaughterhouse was used by several companies and has a processing capacity of approximately 60,000 chickens per day. The beef slaughterhouse has a processing capacity of 40 head of cattle per day. One pork slaughterhouse has a capacity of 1,000 animals per day and the second was located in a suburban area of HCMC and slaughters 200 pigs per day. All chickens and pigs were raised domestically in regions surrounding HCMC, mainly Can Tho City, which is located along the Mekong delta, whereas all beef cattle were imported live from Australia for slaughter. The wholesale fish market was one of the largest in Vietnam, with an average merchandise capacity of 1,000 tons of seafood per day. Among the various types of seafood sold at the market, cultured fish and shrimp were selected for sampling in this study. Nearly all of the meat and fish products processed at these slaughterhouses and wholesale market are transferred to retail supermarkets within HCMC. For the present study, eight large retail supermarkets located in districts 7, 10, and 11 in HCMC were selected for food sampling. For each slaughterhouse, wholesale market, and supermarket, 5–10 food products (>100 g/sample) were randomly selected for sampling. The sampling procedure was performed two to four times in different months (March, June, August, and December). In pork and beef slaughterhouses, lateral muscles of the neck of carcasses were collected as samples. A total of 332 samples were collected from chicken (*n* = 82), pork (*n* = 96), beef (*n* = 72), and fish/shrimp (*n* = 82).

### 2.2. Bacterial Isolation

ESBL/pAmpC-producing* E. coli* strains were detected from food samples using an enrichment procedure [5]. Briefly, approximately 25 g of each sample was incubated in 225 mL buffered peptone water (BPW; Oxoid, Basingstoke, England) at 35°C for 18–22 h. Loopful aliquots from the BPW cultures were inoculated onto CHROMagar ECC plates (CHROMagar, Paris, France) supplemented with 1 *μ*g/mL cefotaxime and were further incubated at 35°C for 22 ± 2 h. Three blue colonies on each plate were picked, isolated, and identified as* E. coli* using conventional biochemical tests, including Triple Sugar Iron slants (Nissui, Tokyo, Japan), Lysine Indole Motility medium (Nissui, Tokyo), and Cellobiose Lactose Indole *β*-Glucuronidase medium (Kyokuto Pharmaceutical Industrial, Tokyo, Japan) [6]. These isolates were subjected to further study.

### 2.3. ESBL and pAmpC Phenotyping and Antimicrobial Susceptibility Testing

ESBL phenotypes were confirmed by the double-disk diffusion test using cefotaxime and ceftazidime with and without clavulanic acid, as recommended by the Clinical and Laboratory Standard Institute (CLSI).

Antimicrobial susceptibility was assessed by the disk diffusion method according to the Performance Standards for Antimicrobial Susceptibility Testing (M100-S23) developed by the CLSI. The antimicrobial agents used in the assay were obtained from BD Biosciences and consisted of ampicillin, cefoxitin, cefotaxime, ceftazidime, meropenem, nalidixic acid, ciprofloxacin, streptomycin, kanamycin, gentamycin, trimethoprim-sulfamethoxazole, tetracycline, chloramphenicol, and fosfomycin. The susceptibilities of ESBL/pAmpC-producing* E. coli* isolates to each antimicrobial agent were categorized as susceptible, intermediate, or resistant according to the CLSI criteria. AmpC phenotypes were estimated based on antimicrobial susceptibility to cefoxitin.

### 2.4. Genotypic Characterization

To screen for the genes responsible for ESBL activity, multiplex PCR analysis was performed for the simultaneous detection of *bla*_CTX-M_, *bla*_TEM_, and *bla*_SHV_ genes. The primers used for PCR amplification were described in a previous study [7].* E. coli* isolates with the ESBL phenotype were classified into the following gene groups: *bla*_CTX-M_ positive and *bla*_TEM_ and/or *bla*_SHV_ positive, but *bla*_CTX-M_ negative. The presence of pAmpC genes responsible for the AmpC phenotype was detected by multiplex PCR, as described by [8]. *bla*_TEM_ genes were identified by sequencing, as previously described [9], and *bla*_SHV_ genes were also identified by sequencing with primers designed in this study (5′-CACTCAAGGATGTATTGTGGTTATGC-3′ and 5′-GCTACGAGCCGGATAACGC-3′).

Phylogenetic grouping of the* E. coli* isolates was determined using a PCR method for three genetic markers,* chuA*,* yjaA*, and* TspE4.C2*, as described by Clermont et al. [10].

To investigate the epidemiological relationships among the ESBL-producing* E. coli* isolates, pulsed-field gel electrophoresis (PFGE) was performed with XbaI (Promega, Japan), as described previously [11]. For the PFGE analysis, five representative strains were selected among isolates of the CTX-M-9 group (phylogenetic group D) having the same phenotypic and genotypic characters, including resistance to the same 9 antibiotic agents. Of the 5 isolates, 4 were isolated from pork (*n* = 2) and beef samples (*n* = 2, block and ground meat) collected from the same supermarket on the same day. The other was isolated from a chicken sample collected from a different supermarket.

### 2.5. Statistical Analysis

Fisher's exact test was used to verify that differences between data sets were significant. A *p* value of <0.05 was considered statistically significant. All statistical analyses were performed using R environment software with the pwr package (js-STAR 2012, http://www.kisnet.or.jp/nappa/software/star/).

## 3. Results

### 3.1. Prevalence of ESBL/pAmpC-Producing* E. coli*

A total of 330 food samples were collected from slaughterhouses, wholesale markets, and supermarkets within the HCMC food distribution network. Strains of ESBL/pAmpC-producing* E. coli* were detected in 150 samples (45.5%). The highest prevalence of positive isolates was in chicken meat (76/82, 92.7%), followed by pork (32/92, 34.8%), beef (18/74, 34.3%), and fish/shrimp (24/82, 29.3%), as shown in Table 1.

Among the samples of chicken meat, ESBL/pAmpC-producing* E. coli* was more frequently detected in the samples obtained from the slaughterhouses (100%) than those collected from supermarkets (85.7%, *p* < 0.05). In contrast, for pork and beef meat, ESBL/pAmpC-producing* E. coli *was more often detected in samples obtained from supermarkets (pork, 50.0%; beef, 40.9%) than in those from slaughterhouses (pork: 22.0%; beef: 0%; *p* < 0.01 and *p* < 0.001, resp.). Among samples of fish/shrimp, no marked differences in the percentages of ESBL/pAmpC-producing* E. coli* detection were found between samples collected from the wholesale market (32.5%) and supermarkets (26.2%) (*p* > 0.10).

### 3.2. Genetic Characterization of ESBL/pAmpC-Producing* E. coli*

A total of 342 strains of ESBL/pAmpC-producing* E. coli* were isolated from 150 positive food samples. The most prevalent* bla* genes responsible for ESBL activity belonged to the CTX-M-9 (110/342, 31.2%) and CTX-M-1 (102/342, 29.8%) groups, whereas the most prevalent* bla* gene responsible for pAmpC *β*-lactamase activity belonged to the CIT group (118/342, 34.5%). Genes of these three major groups were detected in the isolates from all types of examined food, although CTX-M-2, CTX-M-8, and CTX-M-25 group* bla* genes were not detected (Table 2). In addition, isolates containing *bla*_TEM_ genes and either CTX-M-9 (51.8%), CTX-M-1 (74.5%), or CIT group genes (80.5%) were frequently detected. The *bla*_TEM_ genes of 10 randomly selected isolates were sequenced and all were identified as belonging to *bla*_TEM-1_, which encodes penicillinase. Two isolates from chicken samples harbored both CTX-M-1 and CIT group genes. The *bla*_SHV12_ gene was detected in 2 isolates from 2 beef samples and was also found together with the *bla*_TEM135_ gene in one isolate from a chicken sample. The DHA group gene was found in 8 isolates from 6 fish samples, including goby, anabas, swamp eel, red snakehead, and catfish, in addition to one isolate from a chicken sample.

**Figure 1:**
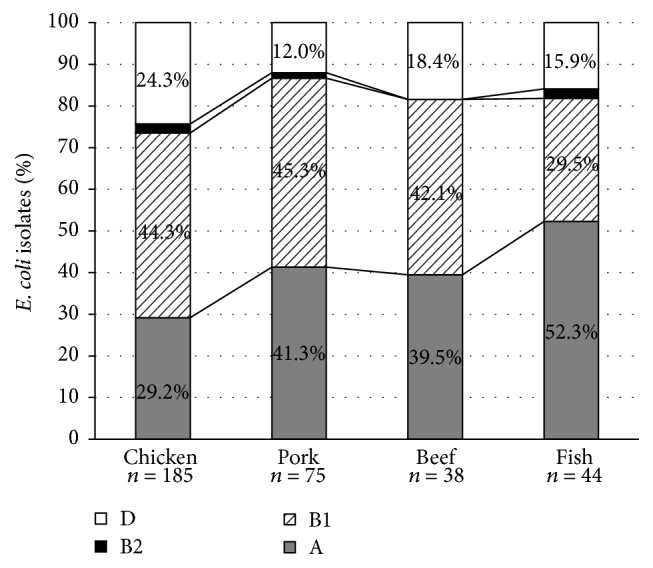
Phylogenetic group distribution of ESBL/pAmpC-producing* E. coli *from food (*n* = 342).

Phylogenetic analysis showed that group B1 (42.1–45.3%) was the most prevalent among isolates from chicken, pork, and beef, whereas group A (52.3%) was predominant among isolates from fish/shrimp (Figure 1). Phylogenetic group B1 was most frequently detected among all isolates (145/342, 42.4%), followed by groups A (123/342, 36.0%) and D (68/342, 19.9%). As expected, phylogenetic group B2 (6/342, 1.8%) was detected at low frequency among isolates from all food types. Although the phylogenetic groups were not linked to definite CTX-M group or resistance patterns, 5 of the 6 isolates that belonged to clinically relevant B2 group [12] were found to contain pAmpC genes.

### 3.3. Antibiotic Resistance Profiles of ESBL/pAmpC-Producing* E. coli* Isolates

The antibiotic resistance profiles of all 342 ESBL/pAmpC-producing* E. coli* isolates are shown in Table 3. High percentages of resistance to ampicillin (100%), tetracycline (94.4%), cefotaxime (88.6%), trimethoprim-sulfamethoxazole (83.6%), streptomycin (83.3%), nalidixic acid (80.4%), and chloramphenicol (79.2%) and a relatively low rate of resistance to ceftazidime (24.6%) were observed among isolates from all food types. In addition, the isolates exhibited high percentages of resistance to ciprofloxacin and fosfomycin but displayed marked variability in resistance between the different food types. In particular, the percentages of resistance to ciprofloxacin and fosfomycin were significantly higher in isolates from chicken (80.5% and 50.8%, resp.) and fish/shrimp (68.2% and 20.8%, resp.) compared to pork (42.7% and 13.3%, resp.) and beef (39.5% and 0%, resp.). Notably, all isolates exhibited susceptibility to meropenem.

**Figure 2:**
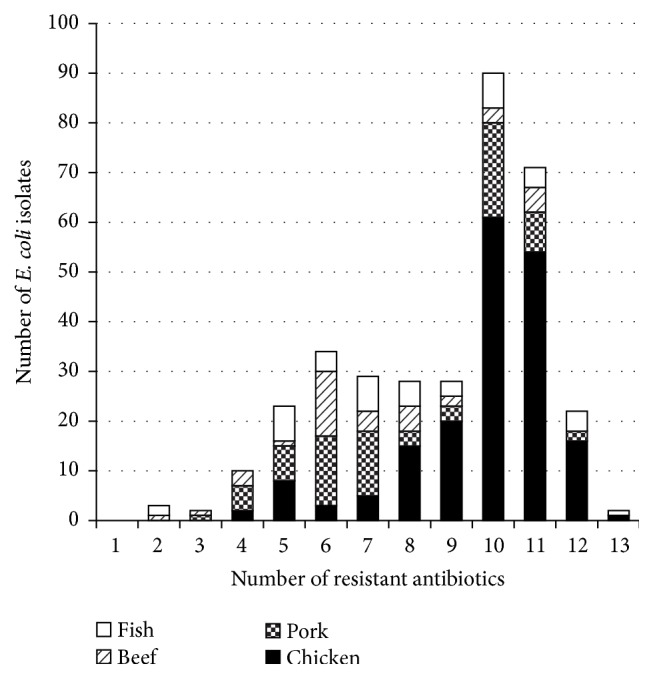
Multidrug-resistance distribution among food isolates of ESBL/pAmpC-producing* E. coli* (*n* = 342).

The distribution of multidrug-resistant* E. coli* isolates from the four food types is shown in Figure 2. Of the 342 isolates, 276 isolates (80.7%) were resistant to at least 6 antibiotic agents and 185 isolates (54.1%) were resistant to at least 10 antibiotic agents. One isolate each from chicken and fish samples was resistant to all 13 tested antibiotics, with the exception of meropenem.

### 3.4. PFGE Profiling

**Figure 3:**
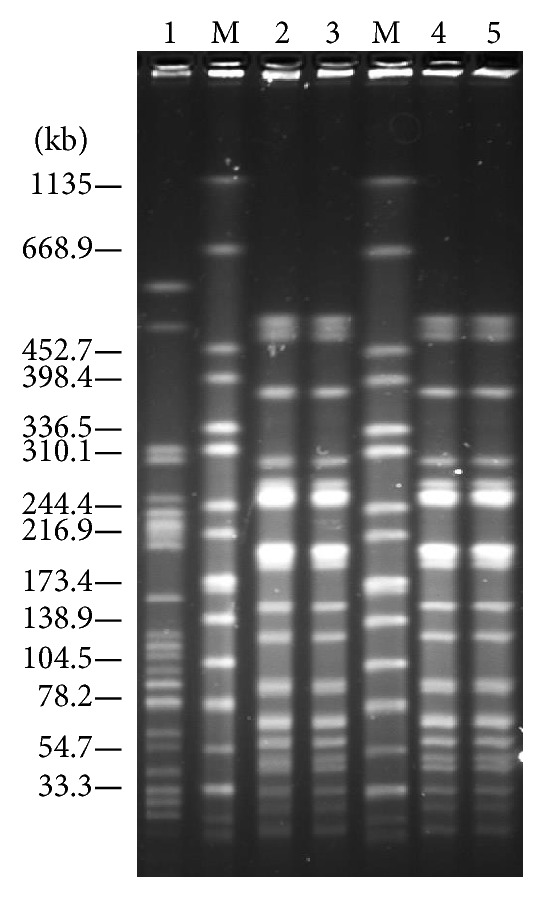
PFGE patterns of ESBL-producing* E. coli* isolates from meat samples in supermarkets. Lanes: 1,* E. coli *isolate from chicken sample collected from a supermarket located in district 10; M,* Salmonella enterica *serovar Braenderup H9812 (a size marker); 2,* E. coli *isolate from a pork block sample; 3,* E. coli *isolate from ground pork sample; 4,* E. coli *isolate from a beef block sample; 5,* E. coli *isolate from ground beef sample; 2–5, isolates from samples collected from the same supermarket located in district 7 on the same day.

The PFGE analysis revealed that ESBL-producing* E. coli* isolates from 2 pork and 2 beef samples collected from the same supermarket on the same day showed an identical PFGE pattern (lanes 2, 3, 4, and 5, Figure 3). In addition, an ESBL-producing* E. coli* isolate obtained from a chicken sample that was collected from a different supermarket showed a distinct PFGE pattern (lane 1, Figure 3).

## 4. Discussion

This study was designed to investigate the presence and distribution of ESBL/pAmpC-producing* E. coli *among animal-based foods and elucidate possible risk factors for the spread of these strains in the food distribution system of HCMC. In particular, the percentages of isolation of these strains from samples collected from slaughterhouses or wholesale markets were compared to those from food samples obtained from supermarkets. The results revealed that the dissemination levels of ESBL/pAmpC-producing* E. coli *at different points in the food distribution network differ among animal-based food. In chicken meat, the highest prevalence of ESBL/pAmpC-producing* E. coli *(100%) was found in samples collected from the slaughterhouse. This result was expected, as two recent studies have shown that healthy chickens at European poultry farms frequently carry ESBL/pAmpC-producing* E. coli* in their rectum (>80% [13] and 72.5% [14]). Thus, significant fecal contamination with ESBL/pAmpC-producing* E. coli* would be expected to occur during slaughter [14]. In particular, fecal bacteria may be readily transferred between chickens via the water-chilling system used in the poultry slaughtering process for aiding in the removal of feathers [15]. The present results suggest that similar cross-contamination events also widely occur in Vietnam and may even be further exacerbated by the lack of sufficient disinfection control practices. Compared to the poultry slaughterhouse samples, the detection rate of ESBL/pAmpC-producing* E. coli *in chicken meat was slightly decreased in supermarkets (85.7%, *p* < 0.05). In the food distribution system in Vietnam, chicken meat is typically cut, deboned, packed, and frozen for sale in supermarkets. It is therefore likely that the freeze preservation process reduces the number of viable bacteria within the meat.

In contrast to chicken meat, a low prevalence of ESBL/pAmpC-producing* E. coli* strains was detected among pork and beef samples from slaughterhouses (22.0% and 0%, resp.). The lower occurrence of ESBL/pAmpC-producing* E. coli* in beef and pork compared with chicken meat has been reported in several countries. For example, ESBL/pAmpC-producing* E. coli *isolates were found in 0%–8% of beef samples, 2%–13% of pork samples, and 15%–95% of broiler meat samples from Sweden, depending on the region of origin [16]. Similar data were reported in Danish [17] and Japanese [18] studies. However, Horton et al. [19] reported high fecal carriage of ESBL-producing* E. coli* in chickens, pigs, and cattle (median levels, chicken: 5,350 CFU/g; pigs: 2,800 CFU/g; and cattle: 100 CFU/g), although the absolute numbers of these resistant isolates and their percentage of the total* E. coli *population varied widely at an individual level. Taken together, these results suggest that the evisceration processes used in beef and pork slaughterhouses have a significant effect on reducing the contamination of carcasses with ESBL/pAmpC-producing* E. coli*. Notably, however, we found that the detection percentages of ESBL/pAmpC-producing* E. coli* in both pork and beef were significantly increased in samples collected from supermarkets compared to those from slaughterhouses (*p* < 0.01 and *p* < 0.001, resp.). In addition, the results of the present PFGE analyses demonstrated that cross-contamination occurred between pork and beef products handled within the same supermarket. The high contamination rate of retail meat with antibiotic-resistant* E. coli* in HCMC was previously reported in pilot studies conducted by Van et al. [20, 21]. The present data indicate that food sanitation practices within the food distribution system of HCMC remain inadequate. To reduce the growth and cross-contamination of ESBL/pAmpC-producing* E. coli* in pork and beef, improved food management practices, such as temperature control and training for food handlers, are urgently required in HCMC.

Our data showed that approximately 30% of farmed fish and shrimp were contaminated with ESBL/pAmpC-producing* E. coli*. The contamination rate was higher than expected based on the reported detection percentages of the bacteria (18.3%) from shrimp in local retail stores in Vietnam [7]. In Vietnamese aquaculture, particularly shrimp farming, the excessive use of antibiotics is a growing concern [22]. The present results suggest that such practices may lead to the development of antibiotic-resistant bacteria.

The findings from several recent reports support the theory that different CTX-M type lineages dominate in different geographical regions. For example, the CTX-M-1 group genes were reported to be the predominant ESBL genes found within isolates from European countries [17], whereas CTX-M-2 and CTX-M-8 group genes have been mainly detected in South America [16] and Japan [18]. Here, CTX-M-9 and CTX-M-1 groups were the most prevalent CTX-M gene types in the meat and fish samples. Rao et al. [23] and Zheng et al. [24] reported that CTX-M-9 group genes were the most frequently detected from animals and food in China, followed by CTX-M-1 group genes. These findings suggest that food chains, including feed and animals, of China and Vietnam are connected, as the two countries are closely related geographically and have shared economic ties for centuries.

To our knowledge, this is the first report of the high occurrence of pAmpC-producing* E. coli* (37.1%) among animal-based foods in Vietnam. With the exception of European meat, few data regarding the prevalence of pAmpC-producing isolates in food are available [16, 17], and considerably fewer reports have been published on the detection of such isolates in humans and food compared to ESBL-producing isolates, although pAmpC *β*-lactamase has been detected worldwide in frequently encountered Enterobacteriaceae isolates, such as* Klebsiella pneumoniae*,* K. oxytoca*,* Proteus mirabilis*,* Salmonella* spp., and* E. coli* [25]. One possible reason for the different percentages of detection is that the growth of pAmpC-producing bacteria is inhibited on specific detection media for ESBL-producing bacteria, such as chromID ESBL (bioMérieux, Marcy l'Étoile, France) and CHROMagar ESBL plates (CHROMagar, Paris, France) [26]. Furthermore, relatively little attention has been paid to the detection of pAmpC-producing bacteria in clinical laboratories because there are currently no CLSI or other approved criteria, including phenotypic confirmatory tests, for AmpC *β*-lactamase [27]. These limitations for detection may lead to underestimation of the prevalence of pAmpC-producing* E. coli*.

Our results show that nearly all pAmpC genes detected in isolates from Vietnamese chicken, pork, and beef belong to CIT group genes, which include *bla*_CMY-2_ gene, a finding that is in agreement with the reported high prevalence of *bla*_CMY-2_ gene in European meat [16, 17]. In addition, DHA group genes, which include *bla*_DHA-1_ gene, were detected among isolates from the fish/shrimp samples at a similar ratio (8/16, 50%) as CIT group genes. The occurrence of DHA group genes was recently reported in clinical and community settings in Netherlands [28, 29]. We also found that DHA group genes were distributed not only among human isolates, but also among isolates from food.

Among the isolates of ESBL/pAmpC-producing* E. coli* detected in the present study, the prevalence of isolates with multidrug-resistance to other classes of antimicrobial agents was particularly high. Approximately 80% of isolates were resistant to at least 6 antibiotic agents, and two isolates from chicken and fish samples were resistant to all tested antibiotics, with the exception of meropenem. In addition, multidrug-resistant ESBL/pAmpC-producing* E. coli* with ciprofloxacin and fosfomycin resistance (80.5% and 50.8%, resp.) was highly distributed in chicken meat in Vietnam, as compared to percentages reported in poultry from Austria (12.3%, ciprofloxacin) [30] and Japan (9.3% and 0%, resp.) [18]. In 2004, 52.6%–63.2% of non-ESBL-producing* E. coli* isolates from Vietnamese chicken meat were reported to be resistant to fluoroquinolones [21]. It is likely that these fluoroquinolones-resistant* E. coli* isolates subsequently acquired ESBL/pAmpC resistance genes in the chicken intestine within the past 9 years.

In the last decade, the incidence of ESBL-producing* E. coli* with multidrug-resistance has significantly increased in food animals in China [23, 24]. In particular, high percentages of resistance to ciprofloxacin, ranging from 79.6% to 88.1% [24, 31], and fosfomycin (30.9%) [23] were reported. Although fluoroquinolones have been widely used in animal farms, fosfomycin has not been approved for use in China or Vietnam. Thus, it remains unclear why fosfomycin resistance percentages among ESBL-producing* E. coli* isolates from animals have remarkably increased in both China and Vietnam. One possible reason for this increase is the coselection with other antibiotic agents, such as cephalosporins, because recent studies have shown that one of the fosfomycin resistance genes,* fos*A3, is colocalized with the *bla*_CTX-M_ gene on the same specific plasmid [32–34].

## 5. Conclusion

This study showed a high prevalence of ESBL/pAmpC-producing* E. coli* isolates among chicken, pork, beef, and fish/shrimp samples collected within the food distribution system of HCMC. These findings demonstrate that animal-based food products in HCMC represent a major reservoir of ESBL/pAmpC-producing* E. coli. *Our data also suggested that food sanitation practices in slaughterhouses and supermarkets have played a major role in the dissemination and growth of ESBL/pAmpC-producing* E. coli* in food. Thus, efforts should be made to control the use of antibiotics for food animals, introduce food sanitation management systems, and monitor for the development and dissemination of multidrug-resistant strains in food in Vietnam.

## Figures and Tables

**Table 1:** Prevalence of ESBL/AmpC-*E. coli* in food obtained from slaughterhouses or a wholesale market and supermarkets (2012 October–2014 March).

Food	Place	Number of samples	ESBL/AmpC-*E. coli*		
			Positive	*p* value^a^	OR^b^, CI^c^
Chicken		82	76 (92.7%)		
	Slaughterhouse	40	40 (100%)	*p* < 0.05	OR = 14.42
	Supermarket	42	36 (85.7%)		CI (0.78–265.07)

Pork		92	32 (34.8%)		
	Slaughterhouse	50	11 (22.0%)	*p* < 0.01	OR = 0.28
	Supermarket	42	21 (50.0%)		CI (0.11–0.70)

Beef		74	18 (34.3%)		
	Slaughterhouse	30	0 (0%)	*p* < 0.001	OR = 0.02
	Supermarket	44	18 (40.9%)		CI (0.00–0.41)

Fish		82	24 (29.3%)		
	Wholesale market	40	13 (32.5%)	*p* > 0.10	OR = 1.36
	Supermarket	42	11 (26.2%)		CI (0.52–3.52)

Total		330	150 (45.5%)		

^a^Fisher's exact test.

^b^Odds ratio.

^c^95% confidence interval.

**Table 2:** Distribution of *β*-lactamase genes in ESBL/AmpC-producing *E. coli* isolates (*n* = 342) from food.

Food	Sampling place	Number of isolates	ESBL genes							pAmpC genes			
			CTX-M-9 group		CTX-M-1 group			SHV12		CIT group		DHA	
			−	+TEM^a^	−	+TEM^a^	+CIT group	−	+TEM^b^	−	+TEM^a^	−	+TEM^a^
Chicken		185	44 (23.8%)		62 (33.5%)			1 (0.5%)		77 (41.6%)		1 (0.5%)	
	Slaughterhouse	111	6	19	0	22	0	0	1	11	52	1	0
	Supermarkets	73	5	14	8	30	2	0	0	1	13	0	0

Pork		75	41 (54.7%)		15 (20.0%)			0 (0%)		19 (25.3%)		0 (0%)	
	Slaughterhouse	31	11	11	3	0	0	0	0	0	6	0	0
	Supermarkets	44	14	5	2	10	0	0	0	3	10	0	0

Beef		38	12 (31.6%)		10 (26.3%)			2 (3.4%)		14 (36.8%)		0 (0%)	
	Slaughterhouse	0	0	0	0	0	0	0	0	0	0	0	0
	Supermarkets	38	9	3	4	6	0	2	0	5	9	0	0

Fish		44	13 (29.5%)		15 (34.1%)			0 (0%)		8 (18.2%)		8 (18.2%)	
	Wholesale market	24	3	2	7	5	0	0	0	2	2	2	1
	Supermarkets	20	5	3	0	3	0	0	0	1	3	2	3

Total (number of isolates)		342	110 (31.2%)		102 (29.8%)			3 (0.9%)		118 (34.5%)		9 (2.6%)	

^a^10 tested isolates harbored TEM-1 gene.

^b^One isolate harbored TEM-135 gene.

**Table 3:** Antibiotic resistance profiles of ESBL/AmpC-producing *E. coli* isolates from food (*n* = 324).

	Chicken	Pork	Beef	Fish	Total
Ampicillin (AMP)	185 (100)	75 (100)	38 (100)	44 (100)	342 (100)
Cefoxitin (CFX)	84 (45.4)	19 (25.3)	14 (36.8)	15 (34.1)	132 (38.6)
Cefotaxime (CTX)	166 (89.7)	68 (90.7)	33 (86.8)	36 (81.8)	303 (88.6)
Ceftazidime (CAZ)	54 (29.2)	10 (13.3)	8 (21.1)	12 (27.3)	84 (24.6)
Meropenem (MEM)	0 (0)	0 (0)	0 (0)	0 (0)	0 (0)
Nalidixic acid (NA)	170 (91.9)	47 (62.7)	25 (65.8)	33 (75.0)	275 (80.4)
Ciprofloxacin (CPFX)	149 (80.5)	32 (42.7)	15 (39.5)	30 (68.2)	226 (66.1)
Streptomycin (SM)	176 (95.1)	52 (69.3)	32 (84.2)	37 (84.1)	285 (83.3)
Kanamycin (KM)	127 (68.6)	32 (42.7)	6 (15.8)	20 (45.5)	185 (54.1)
Gentamicin (GM)	109 (58.9)	44 (58.7)	11 (28.9)	14 (31.8)	175 (52.0)
Trimethoprim-sulfamethoxazole (ST)	164 (88.7)	52 (69.3)	32 (84.2)	38 (86.4)	286 (83.6)
Tetracycline (TC)	176 (95.1)	71 (94.7)	37 (97.3)	39 (88.6)	323 (94.4)
Chloramphenicol (CP)	156 (84.3)	61 (81.3)	27 (71.1)	27 (61.4)	271 (79.2)
Fosfomycin (FOM)	94 (50.8)	10 (13.3)	0 (0)	9 (20.5)	113 (33.0)
